# Characterization of the Leukocyte Response in Acute Vocal Fold Injury

**DOI:** 10.1371/journal.pone.0139260

**Published:** 2015-10-02

**Authors:** Suzanne N. King, Jeremy Guille, Susan L. Thibeault

**Affiliations:** 1 Department of Neurological Surgery, Kentucky Spinal Cord Injury Research Center, University of Louisville, Louisville, Kentucky, United States of America; 2 Department of ENT and Head and Neck Surgery, University Hospital of Pointe Pitre, French West Indies, Pointe Pitre, Guadeloupe; 3 Division of Otolaryngology – Head and Neck Surgery, Department of Surgery, University of Wisconsin-Madison, Madison, WI, United States of America; University of Florida, UNITED STATES

## Abstract

Macrophages location in the superficial layer of the vocal fold (VF) is not only at the first line of defense, but in a place of physiologic importance to voice quality. This study characterizes and compares macrophage function in two models of acute injury. Porcine VF injuries were created bilaterally by either surgical biopsy or lipopolysaccharide (LPS) (1.5μg/kg) injection. Animals were sacrificed at 1- or 5-day post LPS or 3-, 7-, or 23-days post-surgical injury (n = 3/time/ injury). Flow cytometry characterized immunophenotypes and RT-PCR quantified cytokine gene expression. Uninjured VF were used as controls. Post-surgical and LPS injury, SWC9+/SWC3- cells identified as hi SLA-DR+ (p<0.05) compared to controls along with hi CD16+ expression at 1-day and 3-days respectively compared to all other time points (p<0.05). Surgical injuries, SWC9+/SWC3- cells exhibited hi CD163+ (p<0.05) at 3-days along with upregulation in TNFα and TGFβ1 mRNA compared to 23-days (p<0.05). No measurable changes to IL–12, IFNγ, IL–10, IL–4 mRNA post-surgery. LPS injuries induced upregulation of TNFα, IL–12, IFNγ, IL–10, and IL–4 mRNA at 1- and 5-days compared to controls (p<0.05). Higher levels of IL–10 mRNA were found 1-day post-LPS compared to 5-days (p<0.05). No changes to CD163 or CD80/86 post-LPS were measured. Acute VF injuries revealed a paradigm of markers that appear to associate with each injury. LPS induced a regulatory phenotype indicated by prominent IL–10 mRNA expression. Surgical injury elicited a complex phenotype with early TNFα mRNA and CD163+ and persistent TGFβ1 transcript expression.

## Introduction

Acute injuries to the vocal fold lamina propria can cause deformities in the composition of the extracellular matrix (ECM), impairing vibratory function. Macrophages are of special interest in vocal fold, as their strategic position within the most superficial layer of the lamina propria is a location of biomechanical importance to normal voice quality [1]. Recent findings in other respiratory tissue (i.e. lungs) have suggested that macrophages can influence the resolution of inflammation by first provoking early neutrophil infiltration and second, by their capacity to clear apoptotic granulocytes. Determining macrophages involvement in the inflammatory response to vocal fold injury warrants investigation.

Macrophages can polarize into varies phenotypes. Two opposing phenotypic lineages where first described in the literature by Gorden, labeled as classical (M1) and alternatively (M2) activated [2]. Linear theory is based off *in vitro* work demonstrating macrophages ability to change their function in response to local signals in the microenvironment [2, 3]. However, recent *in vivo* work has found a more diverse dichotomy in response to continuously changing environment. To better describe the functional overlap with macrophages, Mosser and Edwards proposed a circular spectrum with three main classifications and several hybrids that merge these phenotypes [4]. In addition to classical activated macrophages, the circular paradigm introduces regulatory and wound healing phenotypes. Classical and regulatory macrophage behaviors both arise in response to infection, but have very diverse inflammatory functions. Classically activated macrophages display cytotoxic features in response to IFNγ and TNFαsignaling by early granulocyte infiltrates and/or autocrine response after toll-like receptor (TLR) ligation. This causes an influx of pro-inflammatory markers (i.e. interleukin (IL)-1β, tumor necrosis factors (TNF)α, interferon (IFN)-γ, monocyte chemotactic protein [MCP]-1, IL–8) inducing chemotaxis and phagocytosis, which can lead to further phagocytic cell infiltration and thus, increase the proteolytic enzymes capable of degrading or altering matrix fibers [5, 6]. On the other hand, regulatory phenotypes are thought to suppress the immune response by secreting high levels of anti-inflammatory cytokine IL–10, which can stabilize IκBα blocking NF-κB activation and reduce neutrophil accumulation. Unlike the classical phenotype, regulatory activation arises following combination of TLR ligation and another stimuli i.e. immune complex, prostaglandins, apoptotic cells. Regulatory macrophages can also produce protease inhibitors and transcription regulatory factors that can reduce TNFα, IFNγ, and IL–1β pro-inflammatory cytokine secretion. Wound healing macrophages are thought to contribute to ECM production, producing high amounts of IL–4 and decreases in IFNγ cytokines. However, there is some discrepancies in the literature regarding the wound healing subtype as inferences were based off injuries induced by pathogens rather than from a blunt trauma [4]. A recent study suggests that wound macrophages exhibit a more complex phenotype involving TGFβ and TNFα signaling rather than IL–4 or IL–13 cytokines [7].

There is a paucity of research regarding specific host response following surgical injury or any other mechanism of injury to the vocal folds. Pathogenic injuries, such as acute laryngitis, activate the host response through TLR binding of the molecular motifs from bacteria cell wall (i.e. lipopolysaccharide [LPS]) with the purpose of eliminating the microbe. Endotoxin challenges stimulate pro-inflammatory factors and neutrophil infiltration, which can differentiate macrophages into classical activated phenotype [8, 9]. Transmuscular surgical injuries to vocal fold, which occur when submucosal or vascular lesions are excised, can obliterate the epithelium, ECM, blood vessels, and lymphatic network that construct the mucosa. Marked expression of IL–1, TNFα, IFNγ, COX2, and NF-κβ has been observed within first 8hrs after surgical injury to vocal fold [10, 11], which is likely in response to byproducts of dead cells (i.e. high-mobility group box [HMGB]-1) and fragmented or altered matrix components at wound site [12]. This coincides with findings of neutrophil-like cells lingering in the wound bed up to three days post injury [13, 14]. Recent studies in other areas of the body have begun to identify unique wound healing macrophage phenotypes in response to sterile surgical injury [15]. Further work is needed to ascertain macrophages involvement in host response after surgical injury to the vocal folds. Analyzing the cell mediated response triggered by DAMP (surgical) or PAMP (LPS) signaling could provide better understanding of the inflammatory characteristics of two commonly seen clinical pathologies in Otolaryngology.

The objective of the present study was to characterize macrophage phenotypes implicated in acute vocal fold injury. First, we hypothesized that macrophages in the lamina propria will express distinct classical and alternative activated phenotypes, depending on the type of acute injury (surgical and LPS induced) and phase of wound healing (i.e. inflammation, proliferation, remodeling). Second, we hypothesized that surgical injury to the vocal fold will lead to the development of regulatory macrophages producing high levels of anti- and pro- inflammatory factors, as well as co-stimulatory markers. We also hypothesis that LPS injury will lead to classical activated macrophages with high expression of pro-inflammatory cytokines and low expression of anti-inflammatory factors.

## Materials and Methods

### Vocal Fold Injury Models

Nineteen female Landrace pigs (8–10 weeks of age; 22–32kg) were sedated using intramuscular (IM) injections of Telazol (2–7mg/kg) and Xylazine (1–2.2 mg/kg) with Atropine (0.05mg/kg), and then intubated with 4.5–5mm endotracheal tube. Animals were placed in dorsal recumbent position and ventilated with Isoflurane (1–2%) for full control of the swallow response. Direct laryngoscopy was completed using a 30° rigid endoscope attached to 988 3 Chip digital camera head and light source (all from Stryker, Kalamazoo, MI), and then projected on to a separate monitor (2000FP UltraSharp; Dell, Plano, TX). Two models of injury were utilized to activate the innate immune cell response to endogenous signals released after surgical trauma (necrotic cells, debris) and exogenous challenge (LPS). Bilateral vocal fold injuries were performed in each animal (2 vocal folds per larynx). Nine animals underwent transmuscular surgical injury bilaterally to the vocal folds by removing a biopsy at the midmembraneous point from the epithelium down to the thyroarytenoid muscle using 2mm cup microforceps. Seven animals underwent bilateral injection of 0.1ml of LPS diluted in 9.0% NaCl saline at ~1.5μg/kg of weight concentration using a 26-gauge needle (Microfrance, Terrebonne, Canada). Optimal LPS dosage was determined for this study, as it was able to produce a moderate inflammatory response while not impeding the airway and/or overly stressing the animals [16, 17]. At induction of sedation, buprenorphine 0.01–0.05mg/kg was provided for analgesia. After 1, 3, 5, 7, or 23 days post injury, animals (n = 3/day) were sedated (as above) and euthanized with an IV injection of Euthasol (1ml/10# weight). Larynges, lungs, and/or blood were collected. Three animals (1 LPS and 2 surgical injury) developed hyperthermia during post-op due to complications related to anesthetic agent and were administered non-steroidal anti-inflammatory drug IV; two of the three animals survived, both were in the surgical injury group. For normal controls, vocal folds were harvested from three naïve pigs weighing ~100kg (similar breed and facility) with no history of laryngeal injury. Preliminary tests were conducted on pigs transferred from another animal protocol that were undergoing terminal procedures. Injured larynges (intubated >4hrs), lymph nodes, tonsils, spleen, and blood samples were collected from transfer pigs. All experimental protocols were approved by the Institutional Animal Care and Use Committee of the University of Wisconsin- Madison.

Our experimental time points for each model represent initial phases of wound healing for each injury condition. Based on our preliminary work, inflammation (i.e. redness, edema) was immediate following injection of LPS to vocal folds and preliminary flow cytometry findings showed a prominent neutrophil-like response up to 8 hrs after administration with low quantity of SWC9+ macrophages found. By one day post, SWC9+ cells were appreciated and therefore, we projected a similar rapid decline in inflammatory state would likely occur within 5 days. As for the surgical injury model, time points were based off of previous work in rat models. Neutrophil response is thought to decline ~3 days post injury, around the same time that levels of reactive oxygen species drops and re-epithelialization occurs [13, 18, 19]. CD68+ macrophages have been shown to be present 7 days post-injury in vocal fold [20], and we estimated that they likely coincide with fibroblasts activity and their ongoing ECM production [21] that is seen through 21 days post injury.

### Immunohistochemistry

To analyze inflammatory cell activity in the tissue, one injured vocal fold from each time point was excised and immediately flash frozen in OCT. Serial coronal sections were taken at a thickness of 6μm per section along the entire length of the vocal fold. Routine processing was performed by re-hydrating sections through series of xylene and graded alcohols. H&E’s were completed to determine morphology and location. Additional sections were incubated with mouse monoclonal MAC387 antibody (1:500; Abcam, Cambridge, MA) at 37°C for 2hrs, anti-mouse Ig secondary with alkaline phosphatase reagent (Vector Laboratories, CA) for 30 minutes, then ImmPACT Vector Red substrate for 20 min, and counterstained with Mayer’s hematoxylin. Appropriate isotype was used as negative control.

### Flow Cytometry

#### Vocal fold Cell Isolation

Immediately following euthanization, tissue samples were harvested and washed 2 times in cold phosphate buffer saline (PBS; Sigma-Aldrich, USA). From each larynx one true vocal fold was dissected for flow cytometry experiments taking care to only remove portions of the cover (i.e. epithelium and lamina propria) with minimal amounts of muscle. Harvested tissue was minced on ice and digested by incubating in a continuous shaker (37°C, 150rpm) with Dulbecco’s (D)PBS containing 0.05% collagenase I, 50U/ml DNase, 10mM HEPES, 5mM Ca+ (all from Sigma-Aldrich) and 2.5U/ml Elastase (Worthington Biochemical Corp, NJ). After 1.5 hours, tissue fragments were dissociated and then incubated at 37°C for 15 minutes. Cell suspensions were further separated with a 70μm filter and cold incubation buffer (RPMI 1640 supplemented with 10% FBS, 0.1% NEAA, 0.1% L-glutamine, 0.1% sodium pyruvate, 10mM HEPES; all from Sigma-Aldrich, USA) was then added to neutralize the collagenase. After centrifuging at 300g for 10 minutes (4°C) and suspending in cold incubation buffer, cell counts were taken using trypan blue exclusion. Our methods isolate ~1.5–8 x 10^6^ total cells per pig vocal fold. Cells were also enumerated from the ventricular folds of each larynx (located just above true vocal fold) and from one lung biopsy using similar methods as described above for use as flow cytometry controls.

#### Cell surface markers

Isotype specific Zenon Mouse IgG labeling kits (Molecular Probes, Invitrogen, OR) were used to label primary antibodies. Following manufactures instructions, primary antibodies SWC9 (mature macrophage), CD16, SLA-DR class II DR, and CD163 (all from AbD Serotec, Raleigh, NC) were pre-labeled with Alexa Flour (A)594, A647, A405, and A430 (Table 1). After five minutes, non-specific IgG was added to block cross labeling. Mixtures were further diluted in PBS and then immediately used. Titration studies were performed using samples from whole blood and injured larynges to determine the appropriate concentrations of each label. The following conjugated anti-pig antibodies were also used simultaneously: CD14 or SWC3 (myeloid cells) labeled to fluorescein isothiocyanate (FITC; Abd Serotec) and human CD152 (CTLA–4) fused to murine IgG2a Fc labeled with RPE (Ancell Corporation, Stillwater, MN), which denotes expression of co-stimulatory markers CD80 and CD86. Isotype specific antibodies were used as controls, and each were labeled as described above.

**Table 1:** Monoclonal anti-pig specific antibodies and their labels.

Specificity	Isotype	Labeled	Flourochrome	Dilution
SWC9 (203A)	IgG1	Zenon	A594	1:50
CD16	IgG1	Zenon	A647	1:50
SLA-DR	IgG2b	Zenon	A405	1:40
CD80/86	IgG2a	Conjugated	RPE	6μl
CD163	IgG1	Conjugated	FITC	10μl
SWC3/CD14	IgG2b	Zenon	A430	1:20

#### Cell Staining

Cells were washed in cold staining buffer (DPBS, 0.1% w/v bovine serum albumin (BSA) fraction V, 1% FBS, 10mM HEPES) and cell suspensions (10^6^ cells/ml) were subsequently aliquoted into polystyrene tubes. Cells were then centrifuged at 300g for 10 minutes to remove supernatant, then 10μl of each labeled antibody was added for 30 minutes at 4°C. Cells were then washed once with staining buffer and then washed an additional two times with PBS. Fixable viability dye eFlour 780 (eBioscience, San Diego, CA) at 1ul/ml concentration was added to exclude dead cells. After 30 minutes, cells were washed three times with staining buffer and fixed using 1.5% paraformaldehyde in PBS for an hour. Fixed cells were filtered with 35μm nylon mesh, centrifuged at 300g x 8 min and then suspended in staining buffer. All incubations were done at 4°C in the dark.

#### Gating Strategy

Data was acquired with LSR II and BD FACSDiva software (BD Biosciences, San Jose, CA) within 24hrs of staining. Single stained samples from whole blood, lung, and ventricular folds were used to define voltage and compensation settings for each experiment. To control for reproducibility, rainbow beads (Spherotech, Lack Forest, IL) were included in all flow cytometry experiments. Data analysis was performed with Flowjo software (FLOWJO, LLC, OR). Due to limited number of cells acquired from each true vocal fold, cells from ventricular folds were used as fluorescence minus one (FMO) and isotype controls to determine gate boundaries. Preliminary tests denoted similar cell distribution and fluorescence intensity between the true vocal fold and ventricular fold. Live cells were identified by inclusion of events negatively expressing dead cell stain. Singlets were isolated using light scatter properties. Macrophage like population was identified by SWC3- and SWC9+ expression and CD16+ and SWC9- identified neutrophil like populations. SWC3-/SWC9+ cells were further enumerated through expression of CD16, CD163, CD80/86, and SLA-DR.

### Gene Analysis

#### Microdissection & RNA Isolation

True vocal folds from two animals were excised and immediately flash frozen in OCT and stored at -80°C for gene analysis studies. Manual microdissection was carried out to provide specific tissue localization at the injury site. Serial coronal sections were taken at a thickness of 30–40μm/ section along the entire length of the vocal fold and slides were immediately stored at -80°C to prevent degradation. Tissue was subsequently stained with Histogene to preserve RNA integrity (Arcturus, Applied Biosystems, Foster City, CA) following the manufacturer’s protocol. Using a stereo microscope, the vocal fold cover was removed from the slide with a scalpel blade immediately following dehydration. Sections were then homogenized by vortexing in lysis buffer. Total RNA was subsequently extracted using Rneasy Mini Kit (Qiagen, Valencia, CA) according to the manufacturer’s instructions. First strand cDNA was synthesized from 1.4 μg of total RNA using an Omniscript Reverse Transcription Kit (Qiagen) and random primers (Integrated DNA Technologies, Coralville, IA). Every 20^th^ section was taken at 6μm thick and subjected to routine hematoylin and eosin (H&E) staining for morphological analysis to determine the area of interest.

#### Polymerase Chain Reaction

To design and test the specificity of each primer pair, single peaks and appropriate sized DNA bands were confirmed with PCR using tissue collected from transfer pigs, including spleen, vocal folds, and tonsils. Primer sequences, gene bank access numbers, and expected PCR product sizes are listed in Table 2. Targets were amplified using GoTaq Hot Start Polymerase (Promega Corporation, Madison, WI) with the PTC- 200 Peltier Thermal Cycler according to the manufacturer’s protocol. Amplifications were optimized for each primer and carried out for 40 cycles as previously described [22]. Yields underwent electrophoresis on 2% agarose gel using ethidium bromide staining for visualization. To establish a standard curve, DNA fragments from vocal fold samples of amplified product were excised from the gel and purified using QIAquick Gel Extraction Kit (Qiagen) following manufacturers protocol.

**Table 2:** Primer Sequences for PCR and RT-PCR.

Label	Seq Name	Forward	Reverse	bp
IL–10	NM_214041.1	CAGGATGACGACTCTACTAAAC	GAAATGGGAGCTGAGGTATC	153
IL–12	NM_214013.1	GGGTGGGAACACAAGAGATAG	TTCATGGACACAGGAACTAACA	94
TNFα	NM_214022.1	CAATGGCAGAGTGGGTATG	CTGGGAGTAGATGAGGTACAG	105
IL–4	NM_214123.1	GCTCTATTCATGGGTCTCAC	GACGAAGTTGCTGGTACAT	75
IFNγ	DQ845173	GATCTGTCACCAAGATCTAACC	GCAGGCAGGATGACAATTA	94
TGFβ1*	NM_214015.1	CGATTAAAGGTGGAGAGAGGACTG	AATGAATGGTGGACAGACACAGG	128
β-Actin	DQ845171	CGGCATCCACGAAACTAC	TGATCTCCTTCTGCATCCT	137
GAPDH	NM_001206359.1	ACACTCACTCTTCTACCTTTG	CAAATTCATTGTCGTACCAG	90
HPRT1	NM_001032376.2	AAGGACCCCTCGAAGTGTTG	CACAAACATGATTCAAGTCCCTG	122

*From published work [35].

#### Real-time Polymerase Chain Reaction

To compare inflammatory gene expression across each time point with both injury models, mRNA levels were measured utilizing relative quantification standard curve approach. SYBR Select Master Mix (Life Technologies) with the Applied Biosystem 7500 Fast System was used to quantify samples. Hypoxanthine phosphoribosyltransferase (HPRT)-1, βActin, and glyceraldehyde 3-phosphate dehydrogenase (GAPDH) housekeeping genes were validated with all samples using RT-PCR. HPRT1 was found to be least variable across all samples and therefore, it was used as the internal reference. Negative (H2O) and positive (spleen) samples were included with each run. Eight-point standard curves were derived for each target in triplicate to quantify gene expression and concentrations were normalized to housekeeping gene.

### Statistical Analysis

Surface marker expression was analyzed in duplicate from 3 animals. Gene expression was analyzed in duplicate from 2 animals. We were interested in comparing the time effects within each injury model. Repeated measures analysis of variance was carried out to test main effects for each assay. LPS and surgical injury models were analyzed separately. If means differed significantly by p-value <0.05, a post hoc Least Squares Means test was performed to compare time effects. p<0.05 was considered statistically significant.

## Results

### Histology Evaluation of Injured Vocal Fold

**Fig 1:**
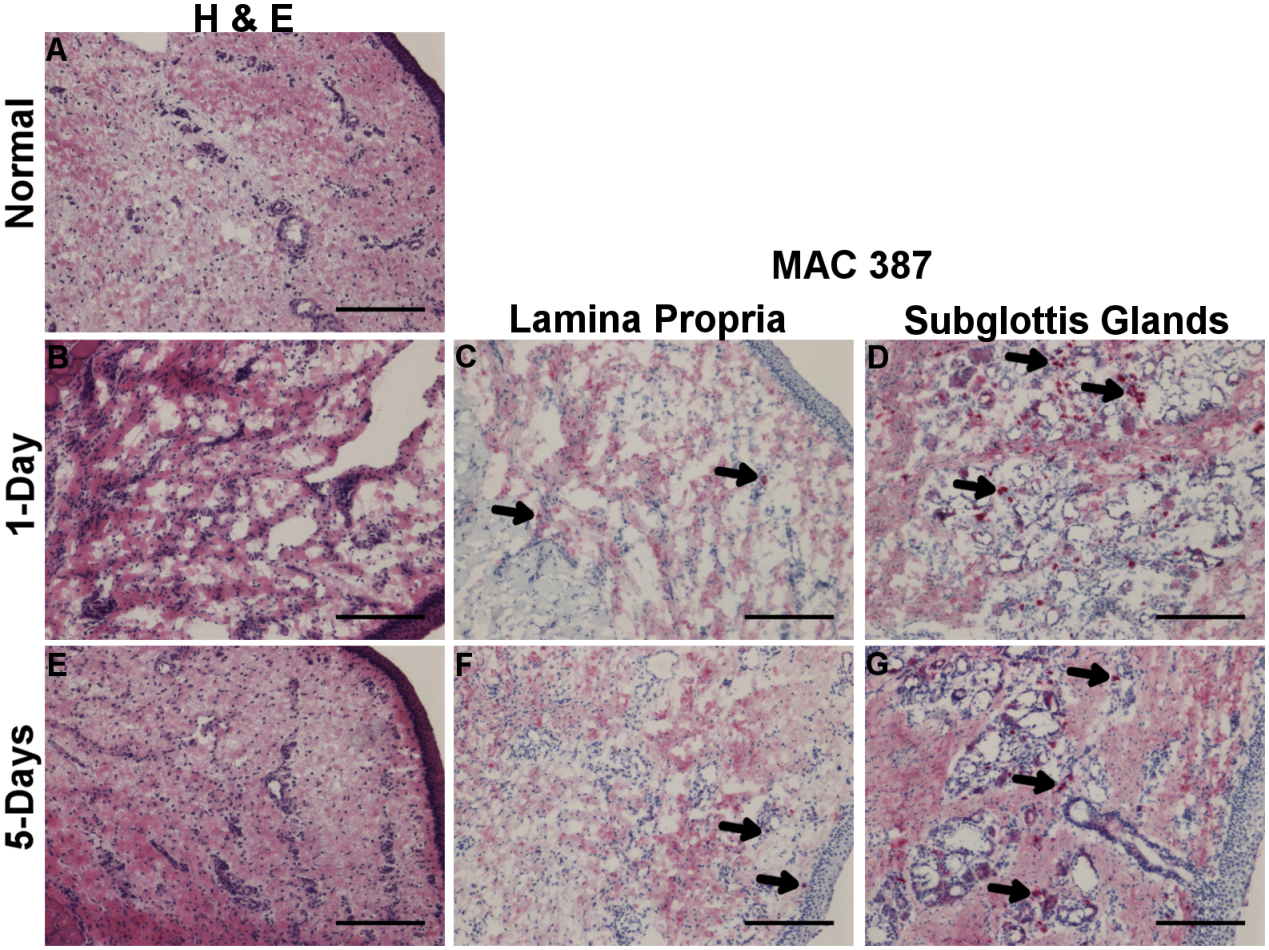
H&E staining 1-day (B) and 5-days (E) post LPS injury and uninjured control (A). Labeling of inflammatory cells with MAC387+ 1-day (C-D) and 5-days (F-G) post LPS injury as indicated by Vector Red staining. Arrow denotes representative positive stained cells. Magnification 10x; 100μm scale bar.

**Fig 2:**
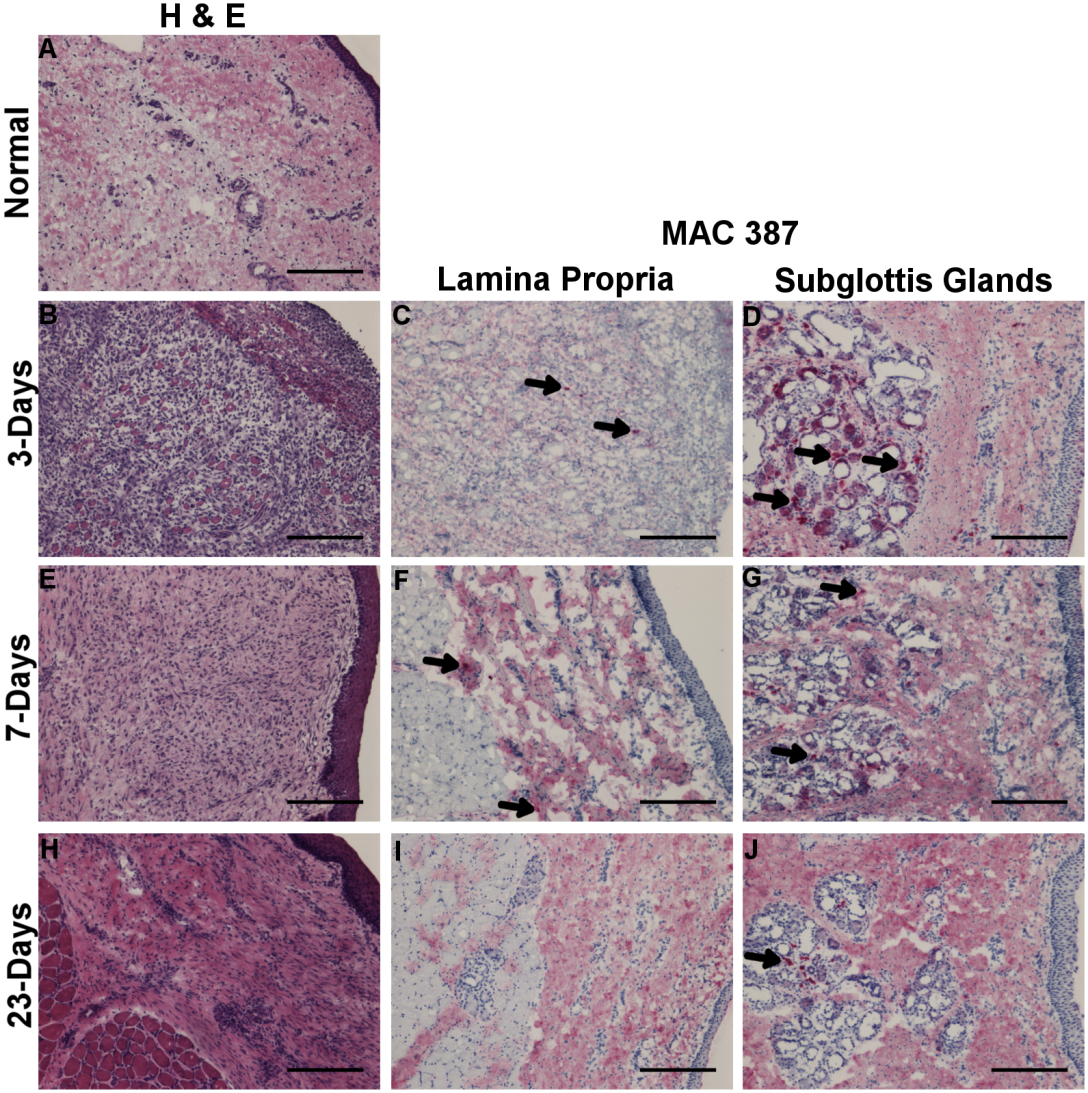
H&E staining 3-days (B), 7-days (E) and 23-days (H) post surgical injury and uninjured control (A). Labeling of inflammatory cells with MAC387 3-days (C-D), 7-days (F-G), and 23-days (I-J) post LPS injury as indicated by Vector Red staining. Arrow denotes representative positive stained cells. Column 1 magnification 10x; 100μm scale bar.

Morphological analysis was conducted using routine H&E staining to determine the area of interest (lamina propria and mid-membranous portion) for purposes of RNA isolation. To analyze infiltration of inflammatory cells into the tissue post injury [23], IHC was carried out against MAC387 surface marker with one vocal fold sample per condition. During an inflammatory reaction, MAC387 (calprotectin) is detectable in infiltrating monocyte derived macrophages and neutrophils; it is not found to be expressed by resident or mature macrophages [24]. Following LPS injury, the total cell density in the lamina propria appeared higher at 1-day and 5-days compared to uninjured vocal fold (Fig 1A, 1B, & 1E). MAC387+ cells were most prominent in the subglottal glands adjacent to vibratory edge of vocal fold after LPS injury (Fig 1D & 1G). Few MAC387+ cells were also apparent across the lamina propria at 1-day post-LPS and by 5-days post they were found in the superficial region (Fig 1C & 1F). Following surgical injury, granulation tissue and disorganization of the muscle fibers is apparent in the wound bed by 3 days (Fig 2B). By 7 days, epithelization and thickening of the lamina propria is appreciated (Fig 2E). Total cell density in the lamina propria appeared higher at 3- and 7-days post surgical injury compared to uninjured tissue. MAC387+ cells were found in subglottal glands similar to LPS injury, although temporal differences were apparent with a gradual decline in number of positive cells at day 23 (Fig 2D, 2G, & 2J). At day 3, few MAC387+ cells are seen scattered throughout the granulation tissue (Fig 2C). By 7-days, couple of MAC387+ cells were found in lamina propria medial to the muscle; staining could be seen in both cytoplasm and around cell infiltrates. After 23 days, no MAC387+ cells were found in lamina propria; however, throughout the lamina propria darker patterns of diffuse staining was appreciated surround a cell nucleus (Fig 2I).

### Vocal fold leukocyte subpopulations

**Fig 3:**
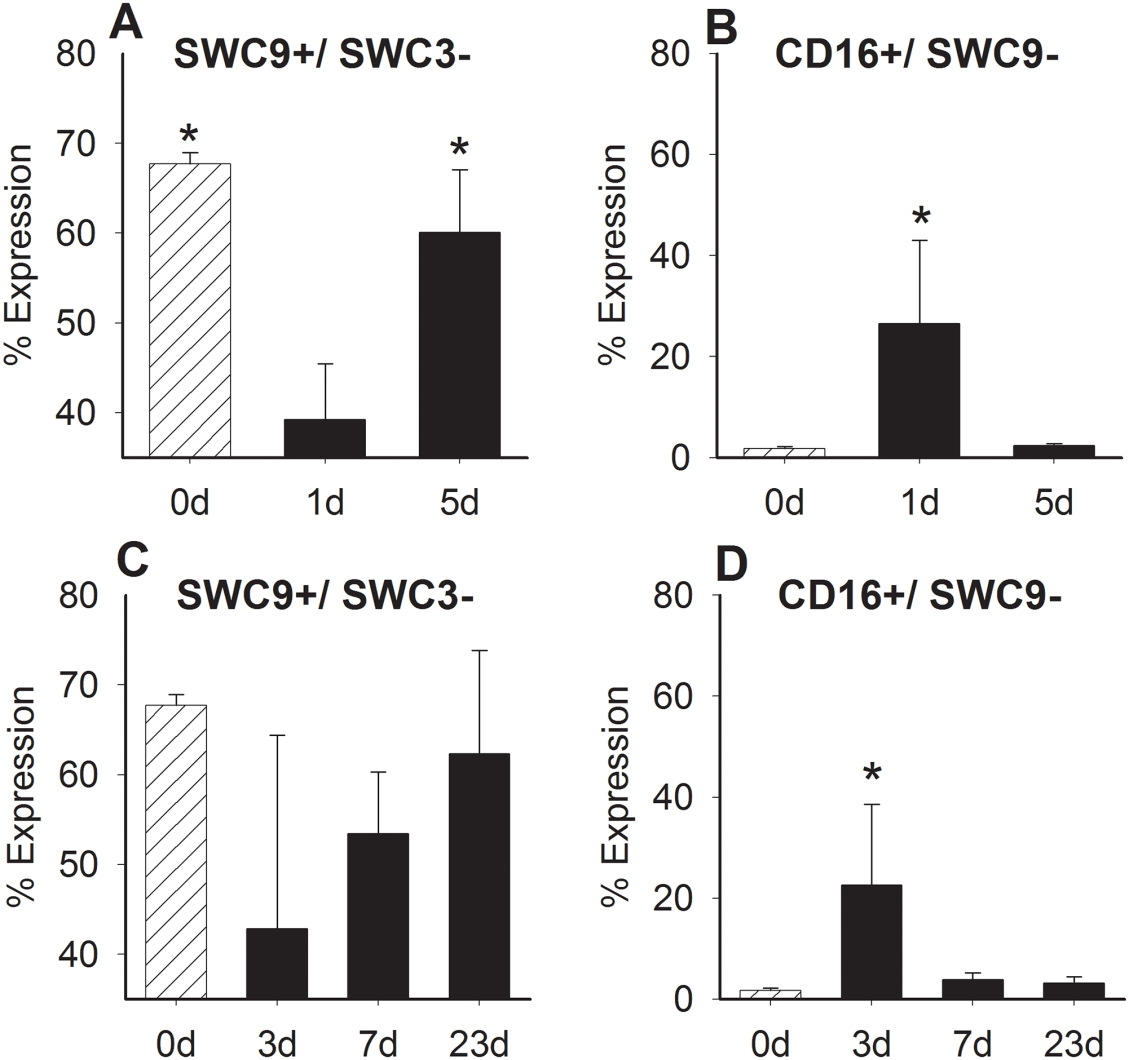
Comparison of SWC9+/SWC3- cells and CD16+/SWC9- cells in the vocal fold lamina propria following LPS (A & B) or surgical (C & D) injury (d = days). Cells were stained for SWC9, SWC3, and CD16. * represents a statistical significance of p<0.05 when compared to other time points.

To determine the immune cell response in the vocal fold following injury, we performed seven-color flow cytometry on cells isolated from the vocal folds using SWC9, SWC3, CD16, CD163, CD80/86, SLA-DR, and dead cell staining. After gating for live and single cells, SWC9+/SWC3- cell and CD16+/SWC9- cell populations were then identified in vocal fold. LPS injury resulted in inverse, re-distribution of macrophage and neutrophil like cells in the tissue (Fig 3A & 3B). The percentage of cells expressing SWC3-/SWC9+ significantly decreased after 1-day in comparison to all other time points (0-day p<0.001; 5-day p< 0.01), while percentage of cells expressing CD16+/SWC9- significantly increased in comparison (0-day and 5-day p< 0.05). In contrast, SWC9+/SWC3- cells concentrations following surgical injury remained practically unchanged from 3 to 23 days (Fig 3C & 3D). However, the percent of CD16+/SWC9- cells was significantly increased at day 3 in comparison to all other time points (p< 0.05).

### Vocal fold Leukocyte Profiles after Injury

**Fig 4:**
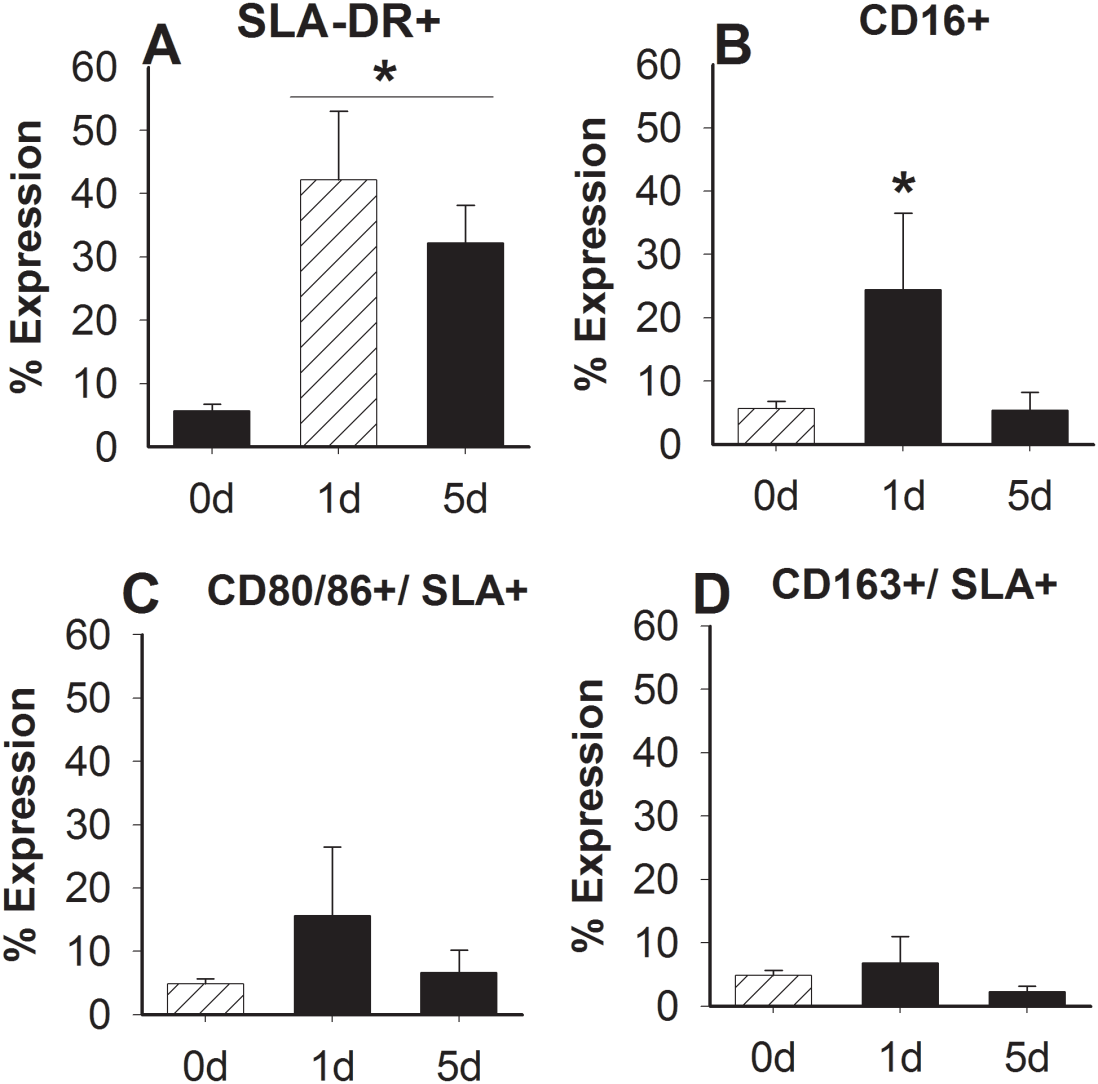
Comparison of surface marker expression after LPS injury. SWC9+/SWC3- cells were further enumerated after 0, 1-, and 5-days (d) by SLA-DR+ (A) and CD16+ (B), as well as SLA-DR+ in combination with (C) CD80/86, or (D) CD163. Data is shown as the mean expression. * represents a statistical significance of p<0.05 when compared to normal control (A) or other time points (B).

To determine phenotypic profile of SWC9+/SWC3- cells after injury, cell surface antigens were measured using combination of CD16+, CD163+, and CD80/86+ with SLA-DR+. Vocal fold SWC9+/SWC3- cells had significantly increased expression of SLA-DR+ after 1-day (p<0.001) and 5-days (p<0.01) post LPS in comparison to non-injured controls (Fig 2A). CD16+ was also found to be significantly higher 1-day post LPS injection in comparison to 5-days post (p<0.05) and non-injured controls (p<0.05) (Fig 4B). The expression of SLA-DR compared to CD16, CD80/86 or CD163 did not change significantly after injection (Fig 4C–4E).

**Fig 5:**
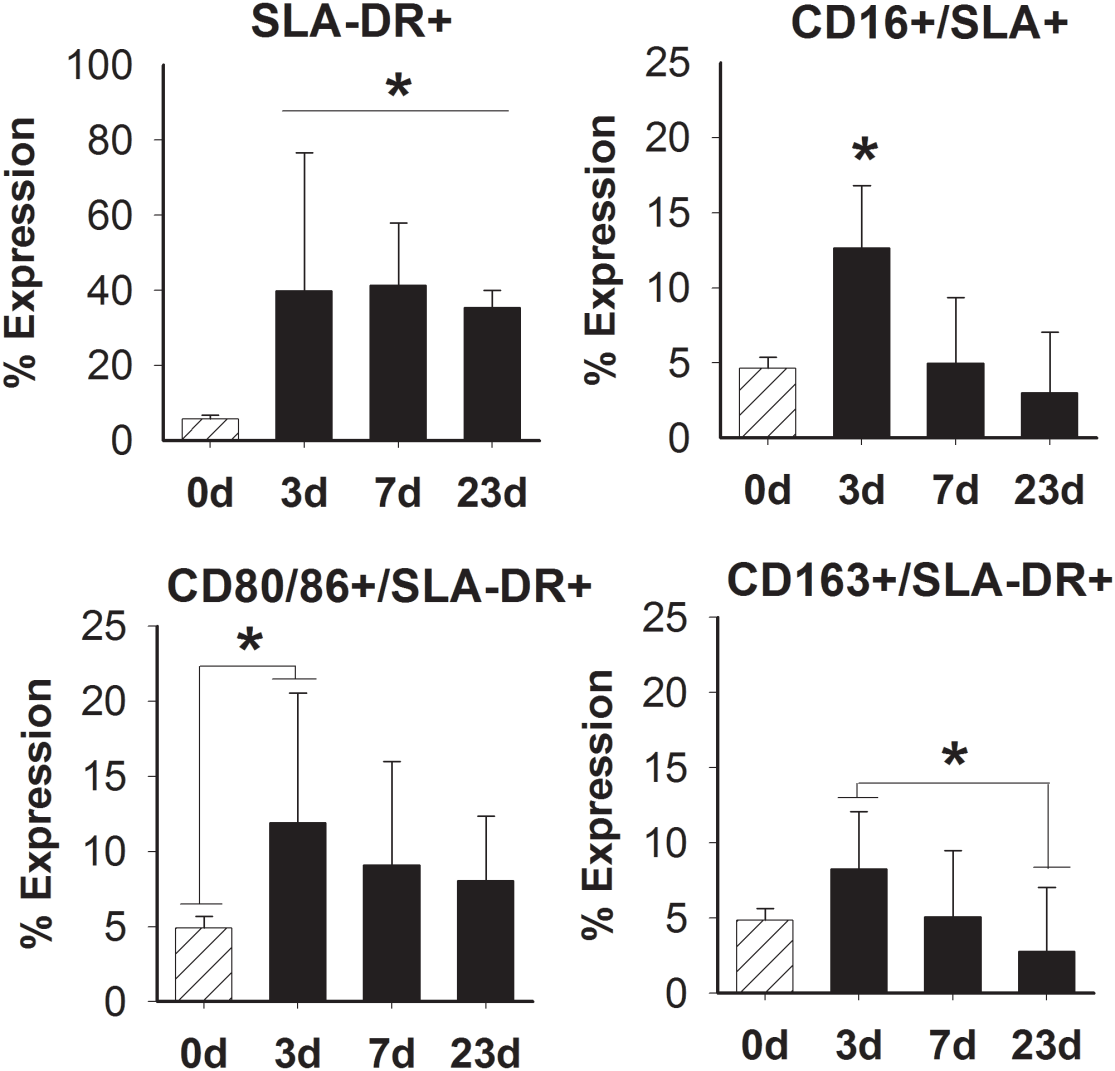
Comparison of surface marker expression following surgical injury. SWC9+/SWC3- cells were analyzed for SLA-DR+ (A) in combination with (B) CD16+, (C) CD80/86, or (D) CD163 at days (d) 0, 3, 7, and 23. Data is shown as the mean expression. * represents a statistical significance of p<0.05 when compared to 0-day or all other time points.

Overall, there was an increased proportion of SWC9+/SWC3- cells producing SLA-DR+ after surgical trauma in comparison to non-injured vocal folds (p<0.0001) (Fig 5A). Significant differences in SLA-DR+ and CD16+, CD80/86+, or CD163+ were found at 3-days post-surgical trauma. CD16+/SLA+ profiles were found to be significantly increased at 3-days in comparison to all other time points (p< 0.001) (Fig 5B). The percent of SWC9+/SWC3- cells expressing CD80/86+/SLA+ was significant after 3-days in comparison to normal control (p< 0.01) (Fig 5C). The expression of CD163+/SLA+ was also increased significantly after 3-days in contrast to 23-days post (p< 0.01) (Fig 5D). No significant changes in surface expression were found between 7- and 23-days post-surgical injuries.

### Inflammatory Profiles after Injury

**Fig 6:**
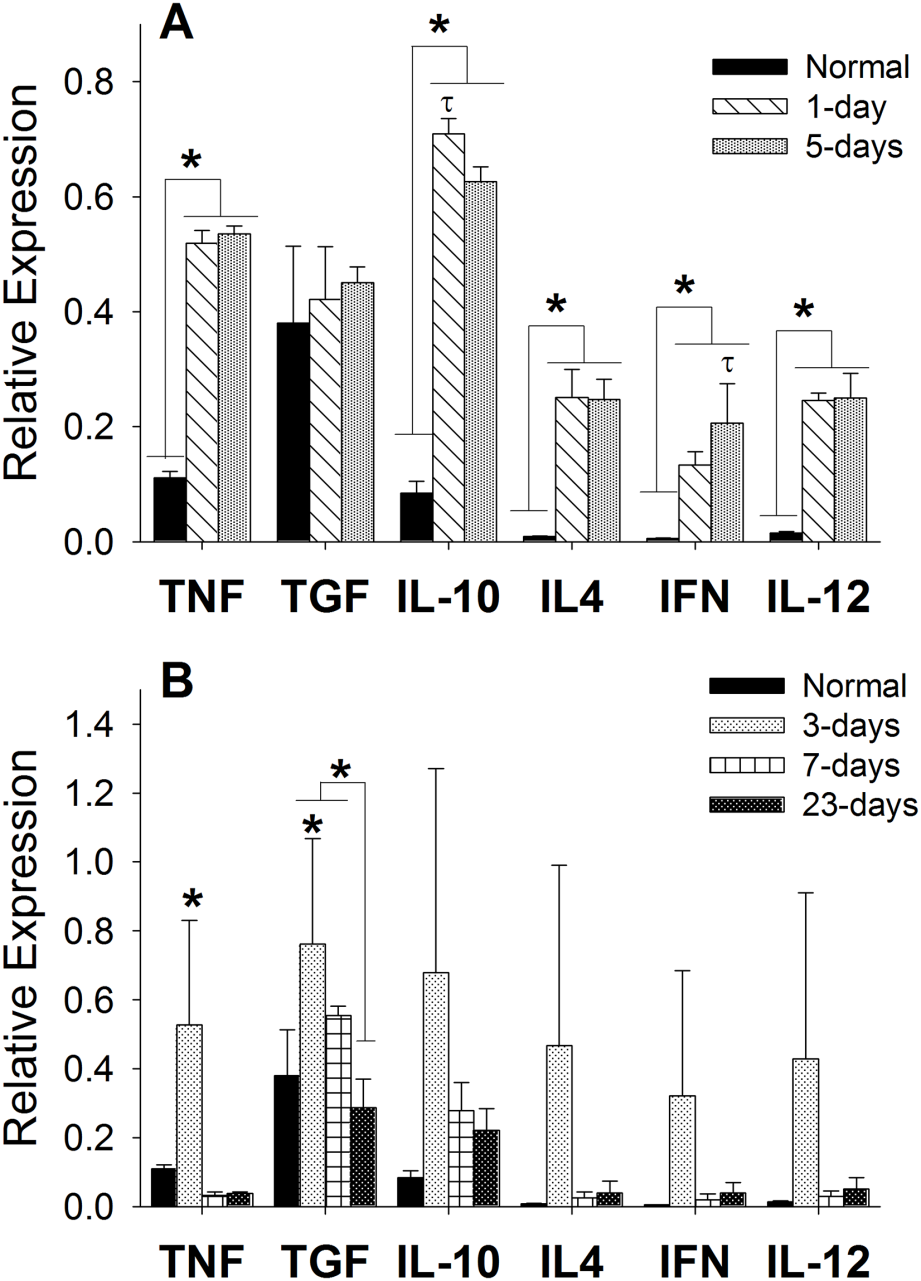
Comparison of gene expression levels following LPS (A) or surgical (B) injury using RT-PCR. Standard curves were derived to quantify gene expression and concentrations were normalized to the HPRT–1 housekeeping gene. Data is shown as the mean expression. A statistical significance of p<0.05 is represented by * when compared to normal control and τ when compared to other time points.

To determine inflammatory profiles after injury, gene expression of TNFα, IL–10, IL–12, IL–4, TGFβ1, and IFNγ was measured using RT-PCR from RNA procured from the vocal fold cover. Following LPS injection, expression of TNFα (p<0.0001), IL–10 (p<0.0001), IL–4 (p<0.0001), IFNγ (p<0.01), and IL–12 (p<0.0001) mRNA was significantly higher after 1- and 5-days compared to uninjured controls (Fig 6A). Statistical differences in IL–10 and IFNγ mRNA expression were found between time points. IL–10 gene expression levels peaked at 3-days compared to 5-days post LPS (p<0.001), where IFNγ expression significantly increased at later time point compared to 3-days (p<0.05). No changes between time points for TNFα, IL–4, or IL–12 were measured. Following surgical trauma, differences in TNFα and TGFβ1 expression were appreciated (Fig 6B). TNFα mRNA expression was significantly highest after 3-days in comparison to all other time points (p< 0.001) and non-injured control (p< 0.01). TGFβ1 expression peaked at 3-days (p<0.01) and 7-days (p< 0.05) post surgery with significant decline after 23-days. Significant increases also noted between 3-days post surgery and non-injured controls (p< 0.01). No changes in IL–10, IL–4, IFNγ, or IL–12 gene expression were found post-surgery.

## Discussion

Injuries to the superficial layer of the vocal fold lamina propria often lead to deformities in ECM composition creating stiffness at the vibratory margin. The objective of this study was to determine underlying macrophage polarizations implicated in acute injury to the vocal folds. We hypothesized that macrophages in the lamina propria will express distinct phenotypic characteristics, depending on the type of injury (LPS or surgery) and phase of healing. Our results demonstrate a paradigm of markers that appear to associate with each injury.

Some commonalities in macrophages phenotype were consistent across injury models. SWC9+/SWC3- cells expressing SLA-DR+ comprised ~40–50% of the cell population found in the lamina propria after LPS or surgical injury across all time points. Strong expression of SLA-DR is often an indicator of high activity in macrophages, suggesting that these cells are increasing their phagocytic efforts and cytokine production [25]. Peak expression of Fc receptor CD16 by SWC9+/SWC3- cells at the earliest time point was also found in both injury models, which may indicate a prolongation of macrophages life span since it inhibits programmed cell death or apoptosis [26]. Interestingly, in normal vocal folds, cells expressing mature macrophage marker (SWC9+) had minimal to no expression of SLA-DR or CD16. It is possible that the initial disturbance from LPS could have activated dormant SWC9+/SWC3- cells residing in the vocal fold. Since surgical injury involves removal of the mucosal, the SWC9+/SWC3- cells found in the vocal fold would have likely been recruited to the injury site. Further work is needed to identify proteomic pattern at the lesion site to help clearly establish the cross-talk between infiltrating and resident immune cells in vocal fold.

Our data does not substantiate our preconception that injecting a single dose of LPS would promote classical activation. Conversely, we found that SWC9+/SWC3- cells from LPS injured vocal folds expressed traits that may be associated with a regulatory phenotype, although patterns of both M1 and M2 paradigms were present. Most notably, responsiveness to LPS resulted in expression of high levels of IL–10 mRNA and modest to low levels of IL–12 mRNA, as well as persistent peaks in pro-inflammatory TNFα mRNA and late upregulation of IFNγ mRNA. It has been suggested that the ratio of IL–10 to IL–12 can define plasticity of activated macrophages [27]. Regulatory macrophages are thought to be distinct from their classical counterpart, due to their potent inhibition of inflammation through high secretion of IL–10 cytokine compared to IL–12 while retaining their ability to produce several other pro-inflammatory markers [28]. The different phenotypic response found in current study may be related to the dose of LPS given, injection delivery method, or differences in the organ. Previous studies in rats have shown that inhaled LPS can induce stages of M1 and M2 cytokine response in the lungs in a dose or time dependent manner as indicated by increases in TNFα expression during early phases and subsequent upregulation of IL–4 later phases [29, 30]. In swine, sensitivity to LPS has been shown to be correlated with the dose and metabolic status of the animal with growing pigs exhibiting increased responses [16]. In the current study, LPS was administered locally and at approximately 3/4^th^ the concentration used in previous swine studies that tested systemic differences to endotoxin challenge at the mid-range level [16, 17]. The efficiency of LPS drainage out of the site of injection can also effect its potency *in vivo*, sustaining the local inflammatory response [31]. The lymph network at the free margin of the vocal fold is limited, however lymphatic flow is likely not affected as breathing and phonation cause ongoing motion that can benefit clearance from the periphery. Vocal fold macrophages response to LPS may be reflective of differences across organs. Fibroblasts are the most abundant cell type in the vocal fold [1] and have been shown *in vitro* to activate TLR4 ligation in response to LPS, although they do not appear to produce measureable levels of TNFα, IL–12 or IL–10 cytokines in response [22]. Further work, has demonstrated that vocal fold fibroblasts can influence the function of activated macrophages when co-cultured together [32]. This may indicate that vocal folds illicit a unique response to endotoxin challenge.

After surgical injury to vocal folds, we found a complex response involving an early production of TNFα mRNA, CD16+, and CD163+ with persistent upregulation in the expression of TGFβ mRNA; along with minimal to no detection of IL–10, IL–12, IFNγ, and IL–4 genes. The phenotype does not appear to conform to profiles described by Gorden’s linear M1/ M2 polarization [2] or Mosser and Edwards circular three classification spectrum, which our hypothesis was based on [4]. These models suggest that induced IL–10 and IL–4 or IL–13 are characteristics of alternative activation or wound healing phenotypes. Although phenotypic classifications are often distinguished based on cytokine production, much of this literature is based off injuries secondary to viral or microbial pathology as opposed to physiologic wounds (trauma).

Our results support the notion that physical injury generates more distinct “wound healing” macrophage phenotypes, likely directed at cleaning up debris and repairing ECM. A recent study in a sterile subcutaneous murine wound (PVA sponge) model reported that wound macrophages underwent mixed phenotypes [15]. Specifically, Daley et al found high mannose receptor and TNFα production, but low or undetectable amounts of IL–10, IL–12, IL–4 and IL–13 [15]. Since transoral vocal fold surgical procedure is not sterile, direct comparison of our model with sterile wounds should be taken with caution. Further work in transgenic macrophage depleted mouse models of wound healing have suggested that TGF and/or VEGF to be key regulators of macrophages wound healing phenotype, as opposed to inflammatory cytokines highlighted in aforementioned studies [33]. Consistent with studies in injured rat vocal folds [10], prominent TGF-β mRNA expression is observed for 7 days following surgical injury. Recent investigation by Chang et al, found sub-populations of CD68+ macrophages expressing TGF-β1 as early as 12-hrs post with the greatest amount of TGF-β1+/CD68+ cells found 24hrs post injury, which gradually returned to baseline 7 days after injury [20]. TGF-β1 is associated with a wide range of effects during wound healing, therefore its persistent expression in current study may reflect ongoing phagocytosis of apoptotic cells, a response to fibrous deposits (i.e. fibrin) that makeup the provisional matrix [34], and/or the mediation of fibroblast matrix activity. Inflammatory cytokines, such as TNFα, IFNγ, IL–10, and various other factors are known to regulate TGF-β1 response through inhibition of myofibroblast differentiation and enhanced disposition of the ECM. Early upregulation of TNFα that was appreciated 3 days post-surgical injury may help regulate early TGF-β1 activity, however by day 7 the inflammatory cytokine cascade quickly drops off allowing for profibrotic activity. Interestingly, increases in the percentage of CD163+ with SWC3-/SWC9+ macrophages were also found 3 days post-surgical injury. CD163 expression has been shown to be inhibited by TNFα and/or TGF-β1 [35]. From our results, it is difficult to dissect the underlying reason for our contradictory findings. It is possible that differences in these signaling mediators is due to prolonged CD16+/SWC3+ neutrophil infiltration who are also prominent expressers of TNFα. Further work is warranted to determine the interplay between neutrophils and macrophages immediately after surgical injury, as well as ascertain the effects of neutrophils enzymatic activity to wound healing.

There are limitations to our study that warrant further discussion. First, from our flow cytometry data there was no way of clearly segregating SWC3 population due to unexpected autoflorescence. This is a common problem with multi-color flow cytometry, which was likely worsened by exposure to proteases in the digestive buffers. It is possible that vocal fold macrophages express SWC3. Secondly, although the surface markers and cytokines used in the current study are commonly used to denote macrophages polarizations in pig, there may be some overlap in their function. The M1/M2 markers (i.e. CXCL’s, nitro oxide) extensively used in rodent models to study macrophages function in vivo, have not been characterized for use in the pig. Future work should utilize microproteomics assays to study patterns of chemokine and cytokine expression in individual cells from vocal fold lamina propria, which will help to determine the time course after injury. Thirdly, our initial time point after surgical injury of 3 days may not have been sufficient for detecting inflammatory cytokines in the pig. Previous reports in rat vocal fold injury models have shown that re-epithelization occurs within 3 days, which is thought to be key marker of neutrophil apoptosis triggering macrophages infiltration [36]. The 3 day time point exhibited the most variability in gene expression and thus, it is also possible that our sample size for RT-PCR studies (n = 2) was not adequate for detecting small differences. Previous reports have exhibited comparable inconsistency at early time points when inflammatory response is highly active [10]. This is likely similar to what is found clinically, as not every patient undergoing a similar vocal fold procedure has similar wound healing responses. Lastly, from our gene analysis data it is difficult to discern which cell such as, macrophages, neutrophils, fibroblasts produced the targeted gene. Attempts were made to analyze cytokine response at the protein level using IHC, however we were not able to accurately measure proteins secondary to non-specific staining.

## Conclusion

LPS injury to the vocal folds revealed a dramatically different phenotype than the surgical injury model. LPS injury appeared to elicit a regulatory macrophage phenotype characterized by expression of SLA-DR+ and high IL–10 mRNA. In contrast, surgical injury appeared to elicit a complex wound healing phenotype characterized by early expression of TNFα mRNA, CD16+, CD80/86+, and CD163+ with more persistent upregulation in the expression of TGFβ1 mRNA.
